# Supplementary material for: Transcriptome Analysis of Early Lateral Root Formation in Tomato
Source: Plants (Basel). 2024 Jun 12;13(12):1620. doi: 10.3390/plants13121620 (PMC11207605; doi:10.3390/plants13121620)
Supplement: Supplementary file 1 [file plants-13-01620-s001.zip › plants-2961937-supplementary/Supplementary data/Supplementary figure.pdf]

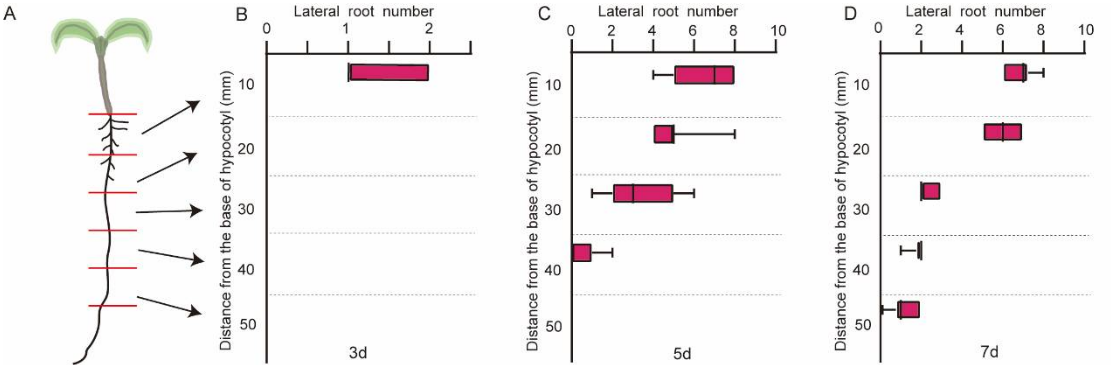

Supplementary FIGURE S1

The number of lateral roots per centimeter in 3, 5, 7d seedlings.

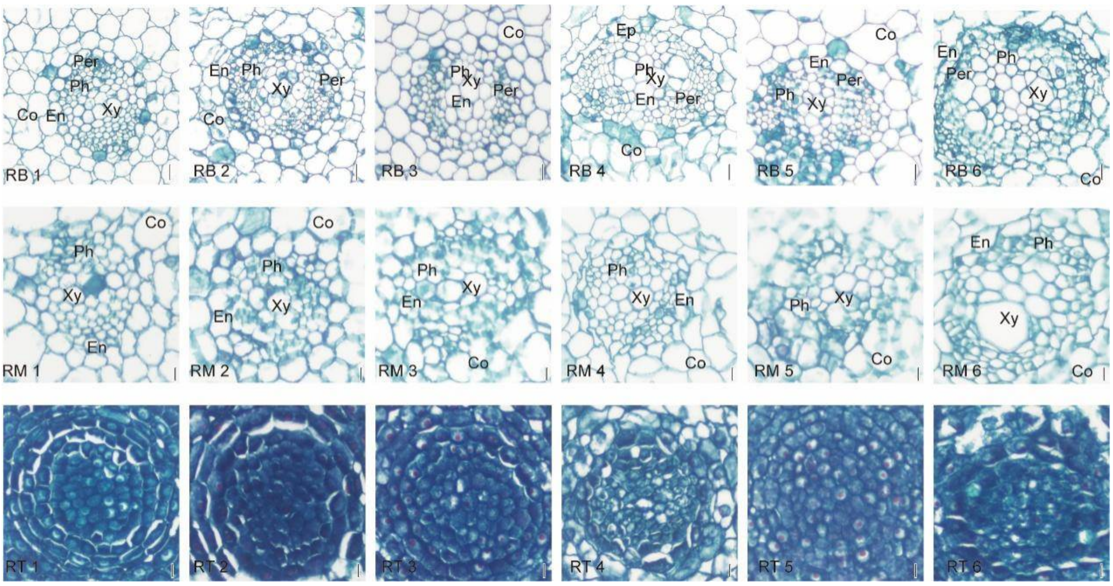

Supplementary FIGURE S2

Cross-section of the root system of tomato seedlings in 1-6 days after sowing. The shootward part of the root (RB), the middle part of the root (RM), and the tip part of the root (RT). Ep, epidermis; Co, cortex; En, endodermis; Xy, xylem; Ph, phloem; Per, pericycle. RB1-6, Bar=150 μm; RM1-6 and RT1-6, Bar=60 μm

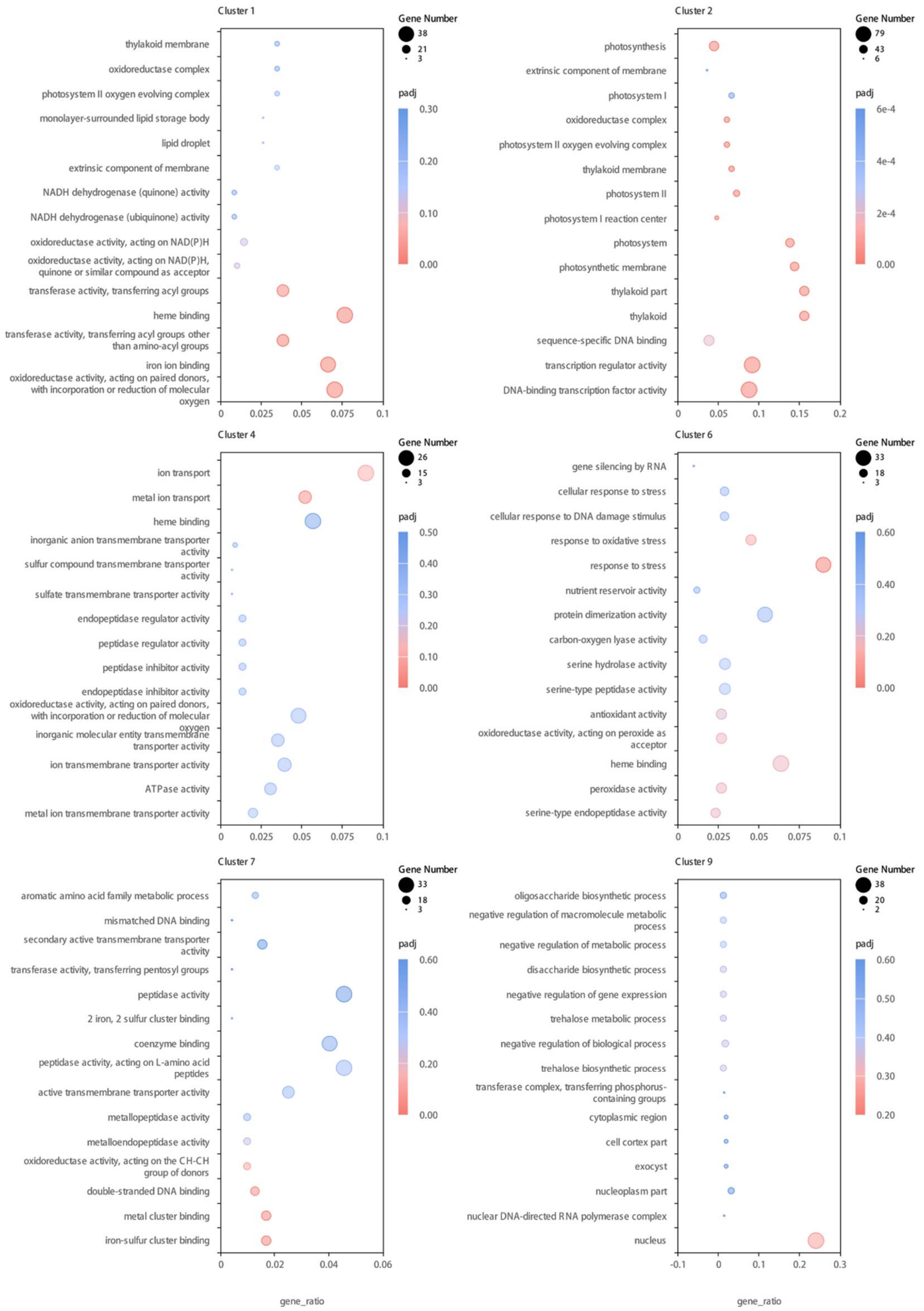

Supplementary FIGURE S3

GO enrichment analysis of gene in Cluster1, 2, 4, 6, 7, 9. For those with more GO terms in the enrichment analysis results, the 15 with the lowest padj were selected for mapping.

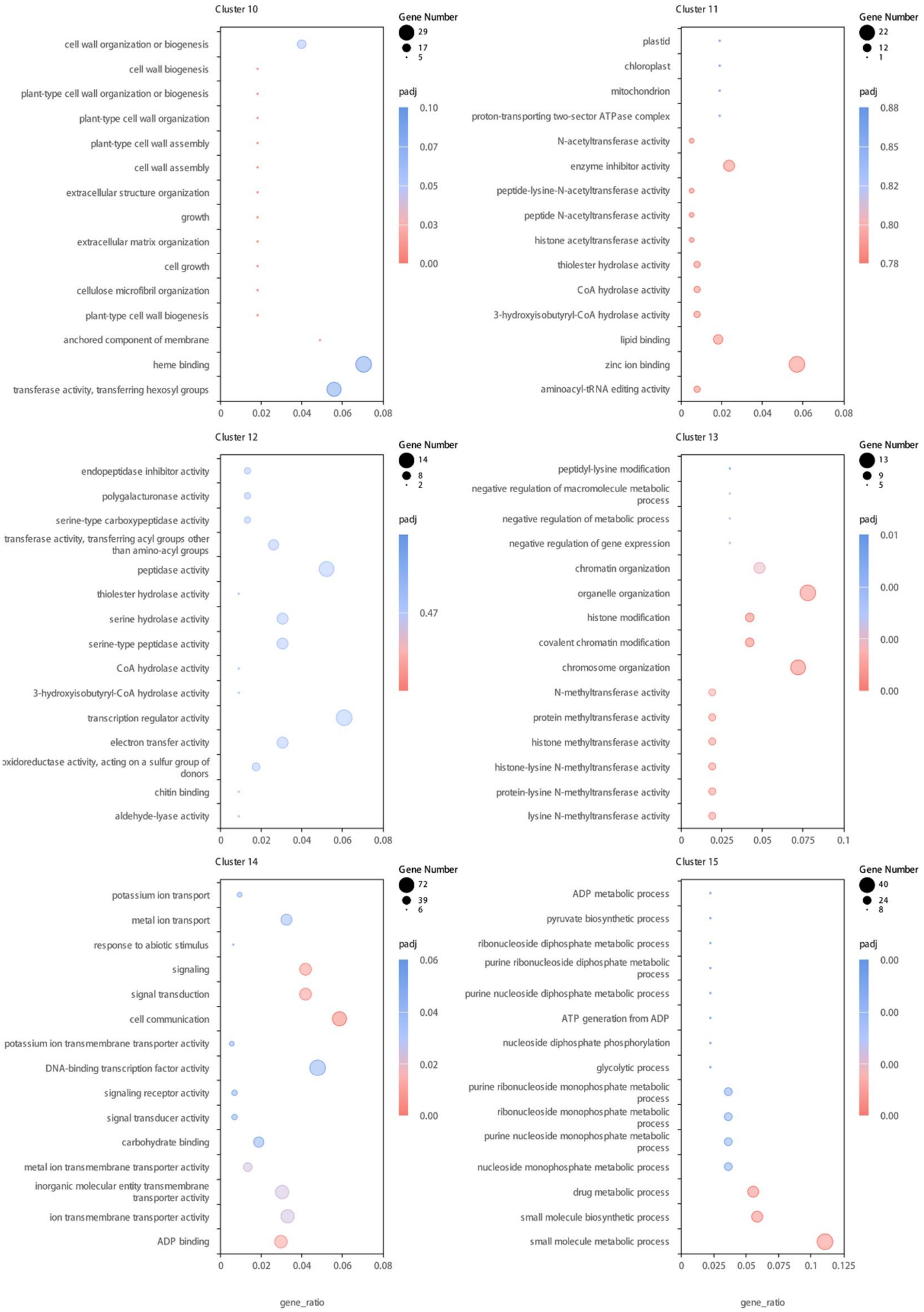

Supplementary FIGURE S4

GO enrichment analysis of gene in Cluster 10-15. For those with more GO terms in the enrichment analysis results, the 15 with the lowest padj were selected for mapping.

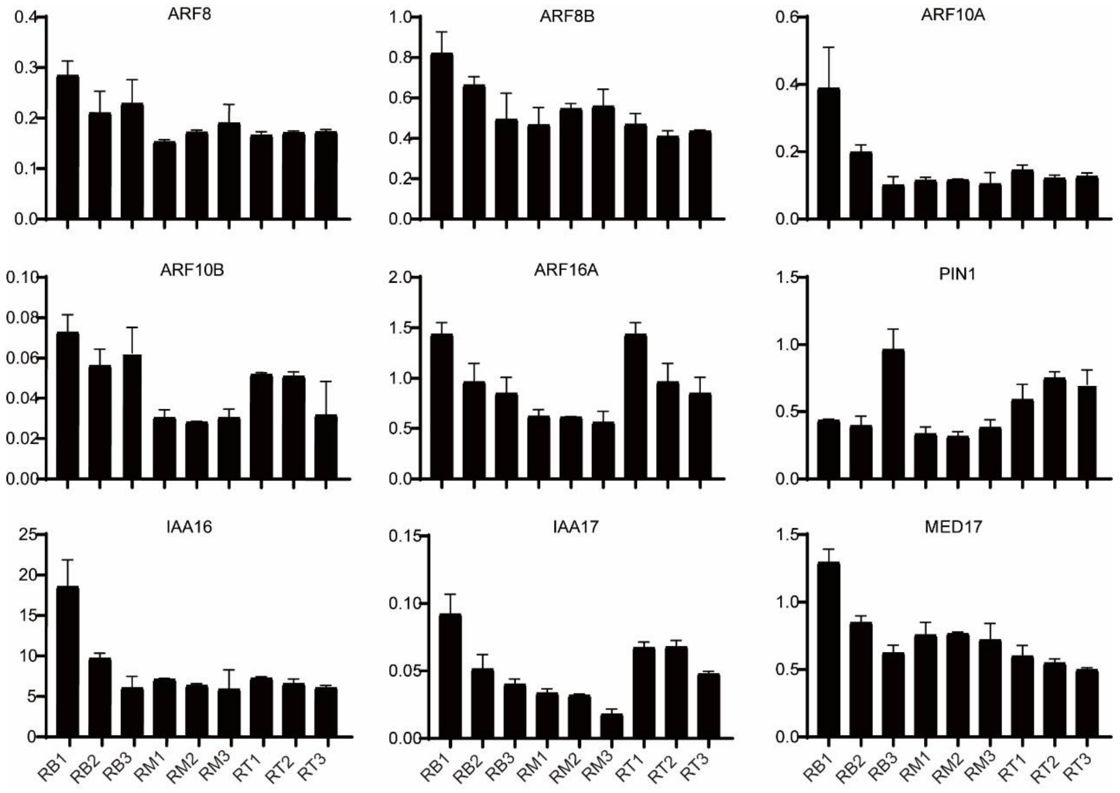

Supplementary FIGURE S5

Expression of 9 maker genes in 27 root samples. Among the 26 genes regulating lateral root development, 9 genes were randomly selected, including auxin response genes ARF8, ARF8B, ARF10A, ARF10B, ARF16A, auxin transport genes PIN1, IAA16, IAA17 and MED17. QPCR analysis was performed on 27 samples of 9 genes.

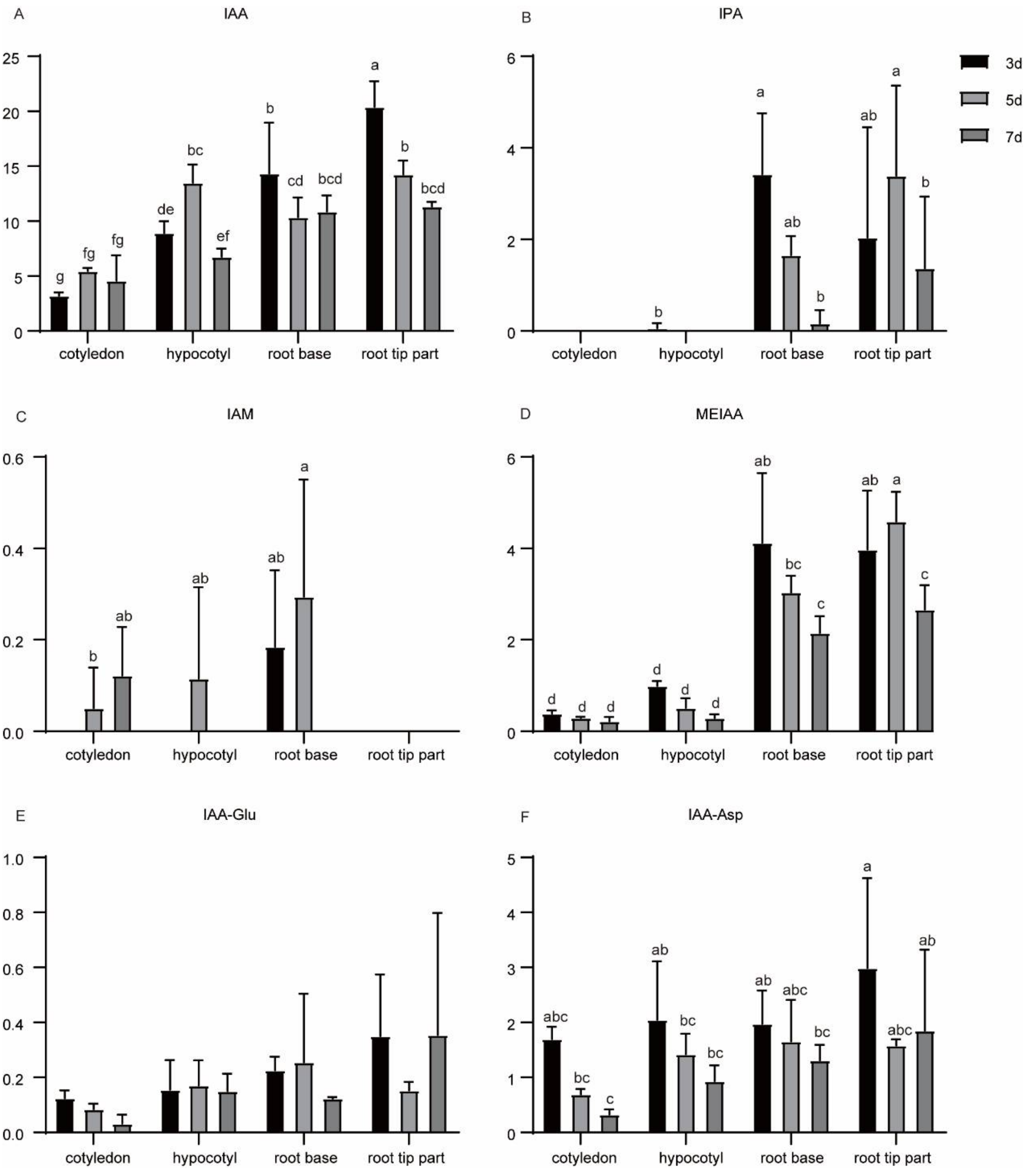

Supplementary FIGURE S6

Auxin content in cotyledon, hypocotyls, root base and root tip part. The black, light gray, brown bars in the figure indicate 3d, 5d and 7d samples, respectively. abcdef in the figure represents significance difference analysis. The same letters indicate no significant difference, while the different letters have significant difference.

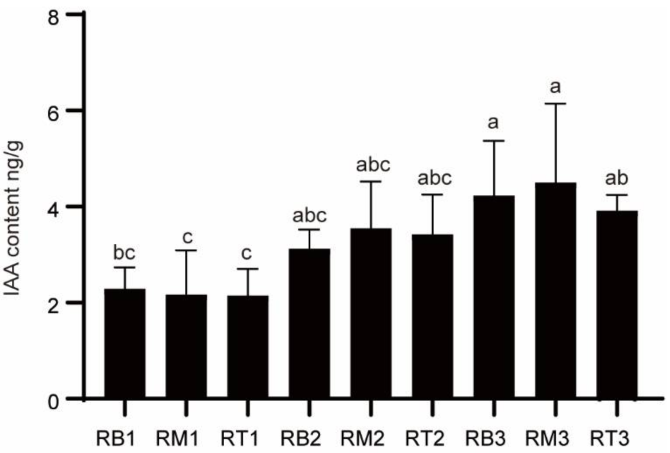

Supplementary FIGURE S7

IAA content in the shootward part of the root (RB), the middle part of the root (RM), and the tip part of the root (RT) at 1d, 2d, and 3d after sowing. abc in the figure represents significance difference analysis. The same letters indicate no significant difference, while the different letters have significant difference.
