# Supplementary figures and images for: Transcriptome Analysis of Early Lateral Root Formation in Tomato
Source: Plants (Basel). 2024 Jun 12;13(12):1620. doi: 10.3390/plants13121620 (PMC11207605; doi:10.3390/plants13121620)

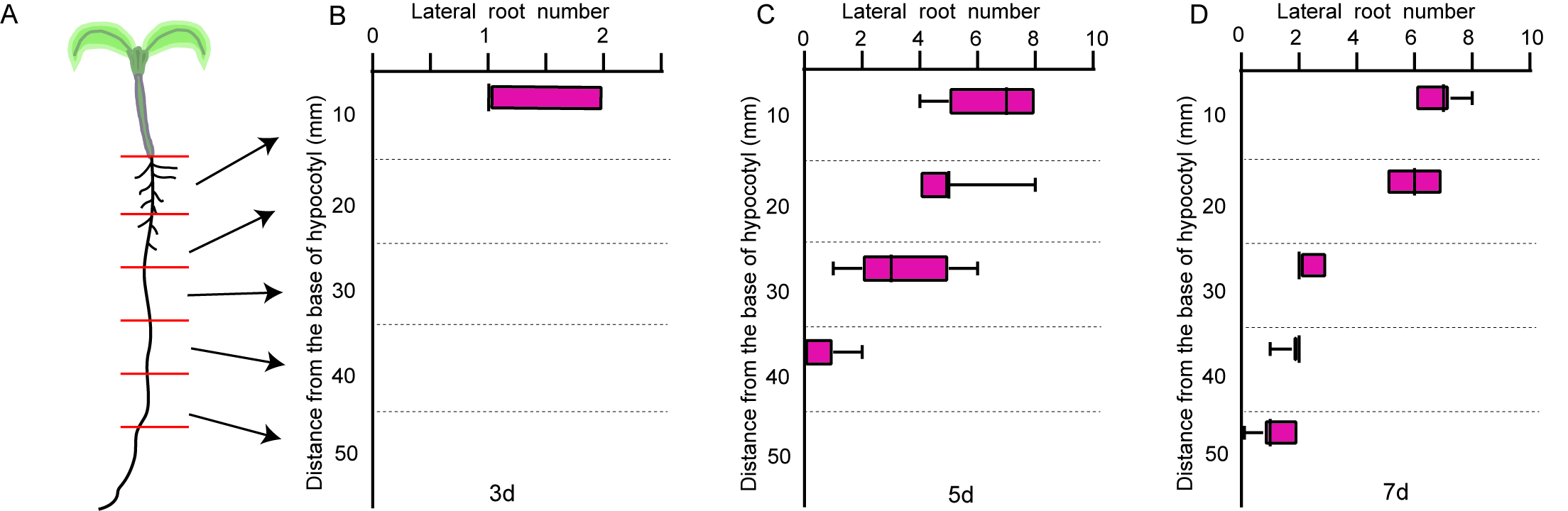

Supplement: Supplementary file 1 [file plants-13-01620-s001.zip › plants-2961937-supplementary/Supplementary data/Supplementary figure/figure S1.tif]

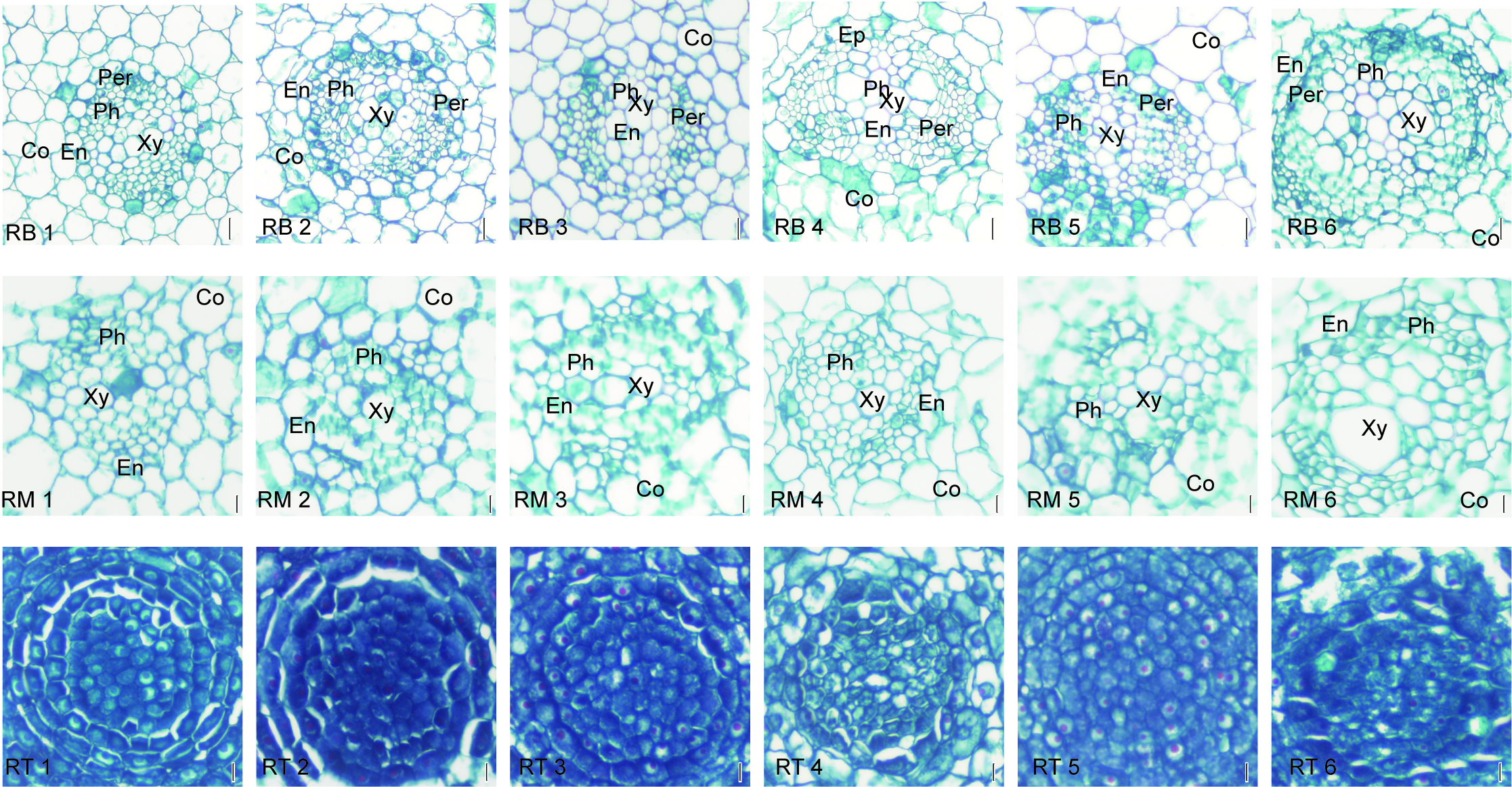

Supplement: Supplementary file 1 [file plants-13-01620-s001.zip › plants-2961937-supplementary/Supplementary data/Supplementary figure/figure S2.tif]

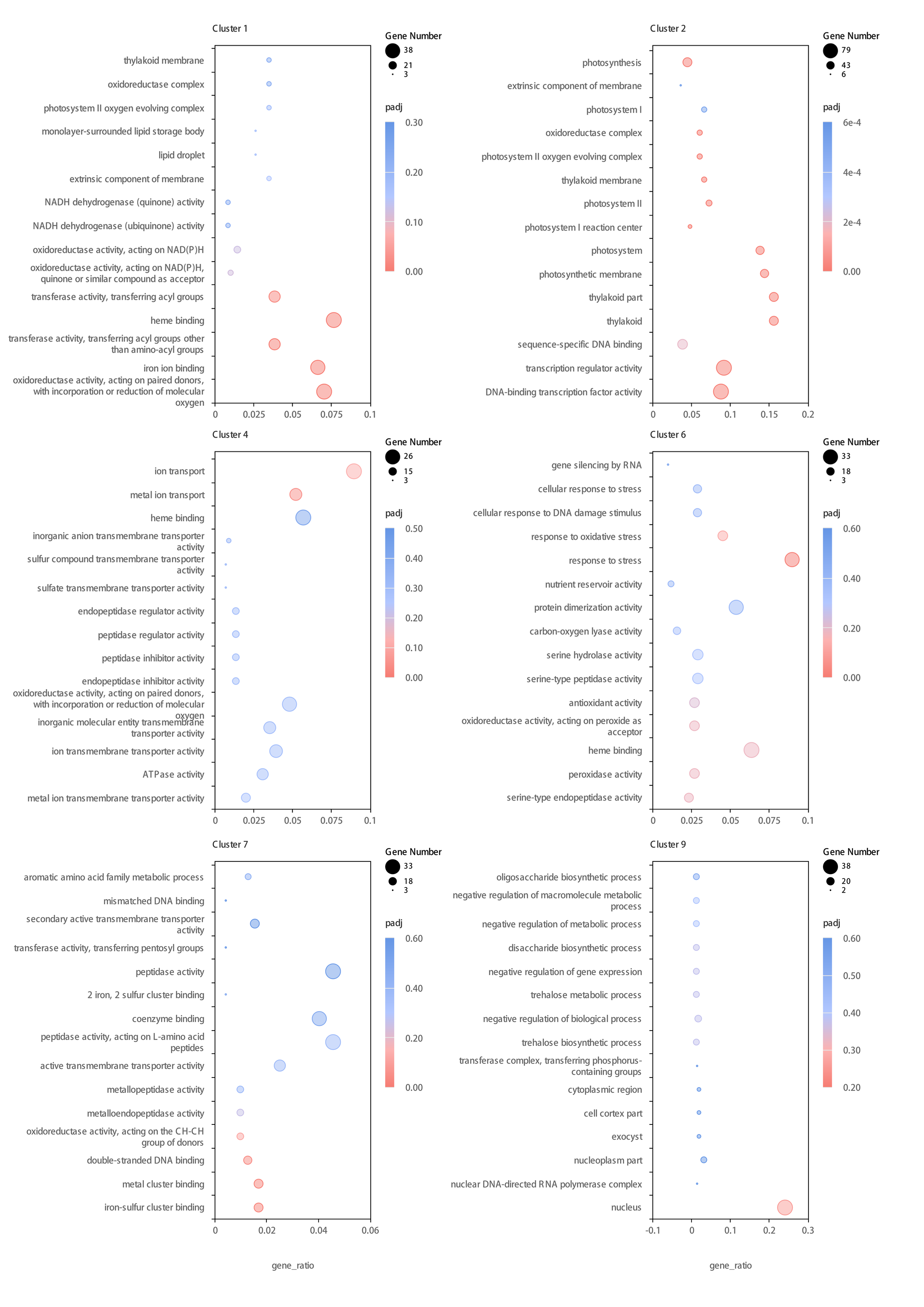

Supplement: Supplementary file 1 [file plants-13-01620-s001.zip › plants-2961937-supplementary/Supplementary data/Supplementary figure/figure S3.png]

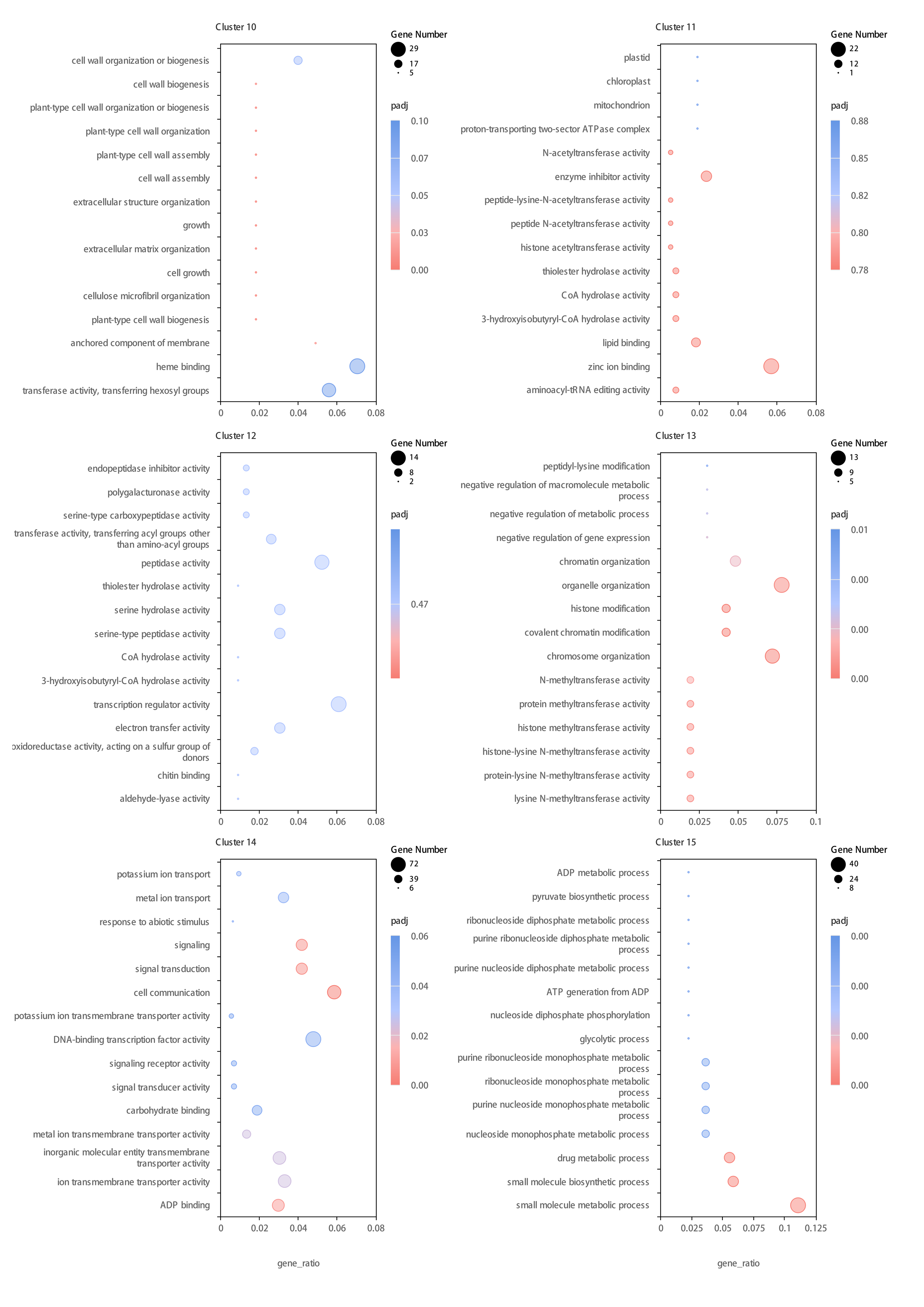

Supplement: Supplementary file 1 [file plants-13-01620-s001.zip › plants-2961937-supplementary/Supplementary data/Supplementary figure/figure S4.png]

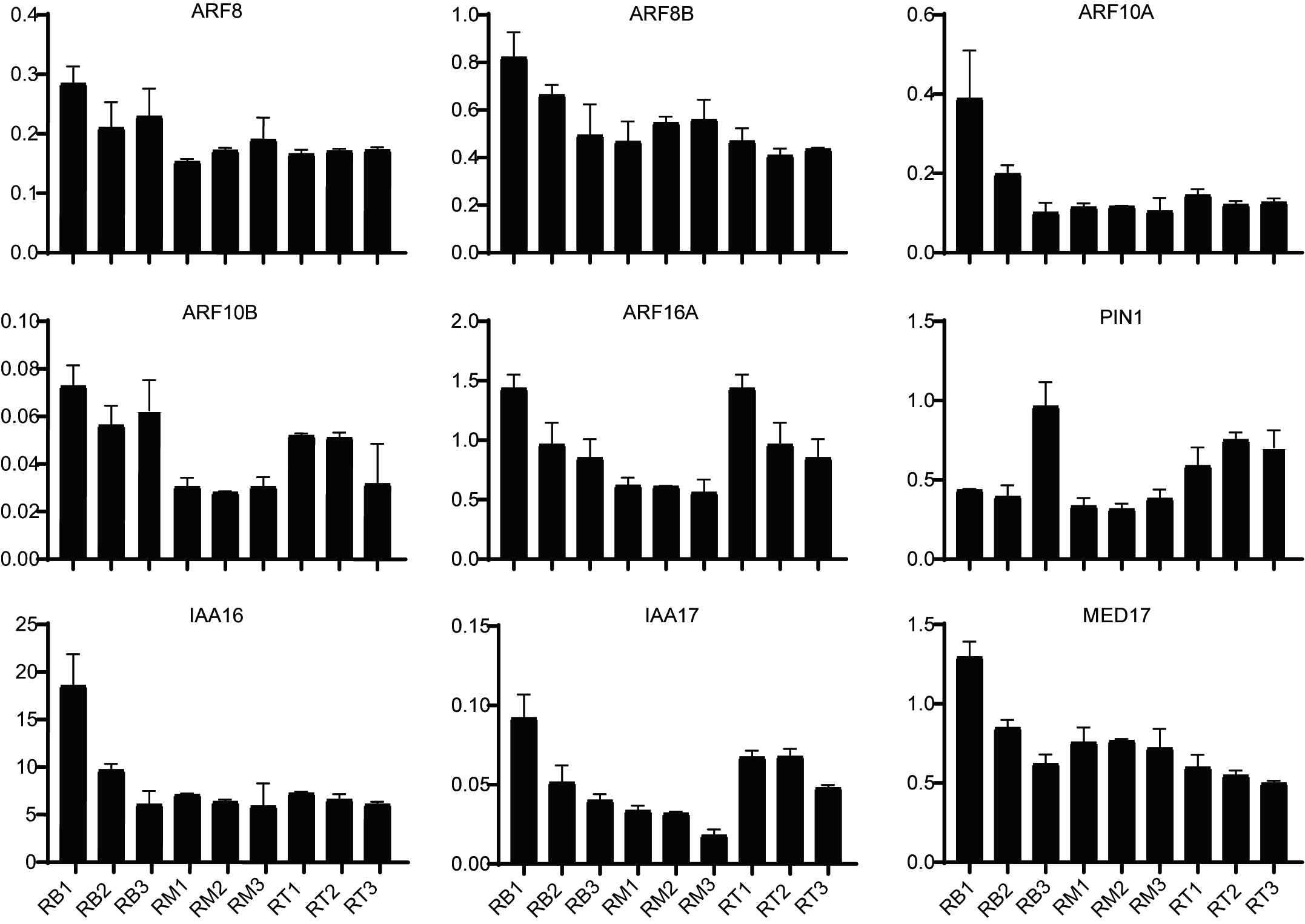

Supplement: Supplementary file 1 [file plants-13-01620-s001.zip › plants-2961937-supplementary/Supplementary data/Supplementary figure/figure S5.tif]

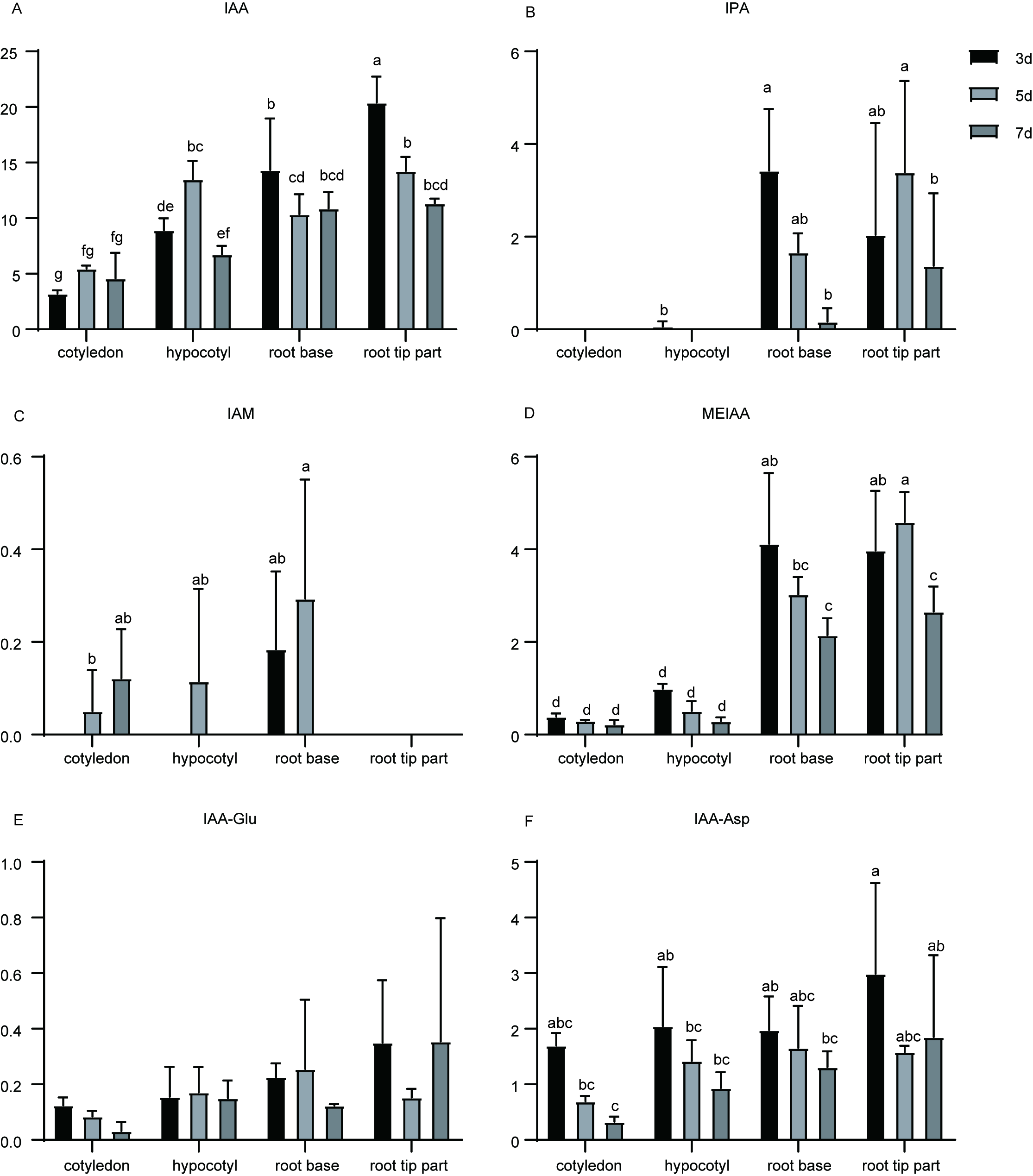

Supplement: Supplementary file 1 [file plants-13-01620-s001.zip › plants-2961937-supplementary/Supplementary data/Supplementary figure/figure S6.tif]

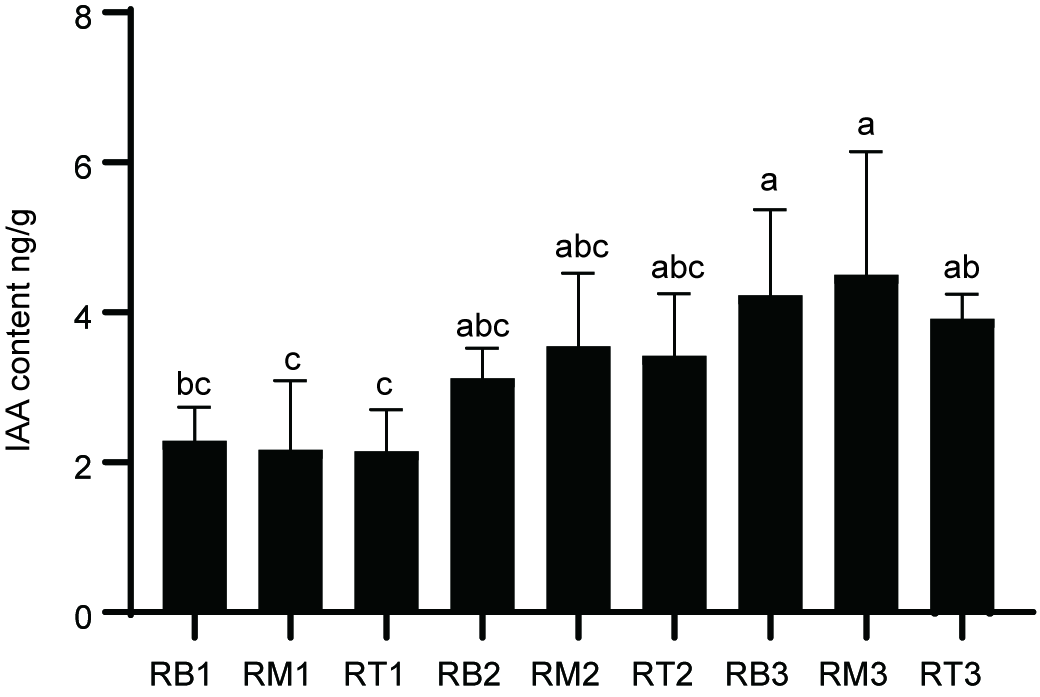

Supplement: Supplementary file 1 [file plants-13-01620-s001.zip › plants-2961937-supplementary/Supplementary data/Supplementary figure/figure S7.tif]
